# Phylogenetic analysis of *pbp* genes in treponemes

**DOI:** 10.3402/iee.v3i0.18636

**Published:** 2013-01-15

**Authors:** Tejpreet Chadha, Adão Alexandre Trindade

**Affiliations:** 1 Department of Biological Sciences, Texas Tech University, Lubbock, TX, USA; 2 Department of Mathematics & Statistics, Texas Tech University, Lubbock, TX, USA

**Keywords:** Treponema pallidum, penicillin-binding proteins (pbp), Tp47

## Abstract

**Background:**

β-Lactamases are the main cause of bacterial resistance to penicillin, cephalosporins, and related β-lactam compounds. The presence of the novel penicillin-binding protein (pbp) Tp47 in *Treponema pallidum* has been reported to be a well-known mechanism for turnover of b-lactam antibiotics. Although, *T. pallidum* remains sensitive to penicillin, clinically significant resistance to macrolides has emerged in many developing countries. The genome sequence of *T. pallidum* has shown the presence of genes encoding *pbp*, but there are no current reports of the presence of mobile plasmids.

**Methods:**

The phylogenetic analysis is used to study the diversity of chromosomal pbp genes and its relatedness to Tp47 in Treponema species.

**Results:**

In our study, genes encoding penicillin-binding proteins that showed significant similarity to each other appeared in separate clusters.

**Conclusion:**

Tp47 showed no substantial similarity to other β-lactamases in treponemes. The relatedness of *Treponema denticola* to other treponemes, including *T. pallidum*, and the reported presence of natural mobile antibiotic determinants highlight the importance of investigating the diversity of *pbp* genes in *Treponema* species. This will lead to a greater understanding of its potential to develop additional antibiotic resistance via horizontal gene transfer that could seriously compromise the treatment and control of syphilis.

The spirochaetes belong to the phylum of gram-negative bacteria—long, 0.1–0.5 µm in diameter, helically coiled, axial flagella that run lengthwise between the bacterial inner membrane and an outer membrane in the periplasmic space (1). Spirochetes are widely distributed in nature, typically anaerobic and free living, though many are notorious parasites. They are commonly isolated from the human mouth, marine environment, ulcerative lesion of a bovine foot, and the surfaces of protozoa that live in termite guts. *Treponema pallidum*, the most investigated treponemes, causes the sexually transmitted disease syphilis. The non-venereal treponemal infections include yaws, bejel, and pinta—endemic to remote regions of Africa, Southeast Asia, and South America (2).

**Table 1:** Summary of pathogenic and saprophytic *Treponemes*

Taxa and strain information	NCBI Taxon ID
*Treponema azotonutricium ZAS-9*	545695
*Treponema brennaborense DD5/3, DSM 12168*	906968
*Treponema denticola ATTC 35405*	243275
*Treponema pallidum pallidum Nichols*	243276
*Treponema pallidum pallidum SS14*	455434
*Treponema paraluiscuniculi Cuniculi A*	545776
*Treponema primitia ZAS-2*	545694

The genus *Treponema* has further expanded to include treponemes with new ecological niches. Their role in the ecosystem is not completely understood, as three-fourths of the treponemes have not been sequenced. This limitation lasts as treponemes have largely proven difficult to culture. The 16S rRNA gene sequences have been the only way to identify oral *Treponema* species (3–7). The recent advancement in culture and sequencing techniques has helped to identify and characterize several *Treponema* species. *Treponema paraluiscuniculi* are known to infect rabbits; *Treponema denticola* is associated with periodontal disease; *Treponema bryantii* are isolated from bovine rumen; *Treponema primitia* and *Treponema azotonutricum* have been isolated from the termite hindgut. *T. bryantii* and *Treponema saccharophilum* have been isolated from the rumen of cows, while *Treponema succinifaciens* have been isolated from swine. In the gastrointestinal tract of termites, comparative 16S rRNA gene sequence analyses have revealed more than 67 different treponemal phylotypes (3, 4, 8). The research study by Lilburn et al. reported that spirochetes from termite hindguts and freshwater sediments possess homolog of a nitrogenase gene (*nifH*) and exhibit nitrogenase activity (8, 9). This observation suggests that the spirochete symbionts have probably coevolved within specific termite species. The ectosymbiotic and free-swimming spirochetes play an important role in metabolic interactions within the host and benefit each other. The motility of spirochetes allows them to occupy unique ecological niches, such as the guts of certain arthropods and the rumen of cows and sheep (4, 10, 11).

**Table 2:** Strains with accession numbers used in this study

GenBank, accession no.	Bacterial strain
Gene ID: 2611355	*Treponema pallidum pallidum Nichols*
Gene ID: 6333587	*Treponema pallidum pallidum SS14*
Gene ID: 10884451	*Treponema paraluiscuniculi Cuniculi A*
Gene ID: 2739572	*Treponema denticola ATTC 35405*
Gene ID:10681574	*Treponema primitia ZAS-2*
Gene ID: 10580463	*Treponema brennaborense DD5/3, DSM 12168*
Gene ID: 10678178	*Treponema azotonutricium ZAS-9*
Gene ID: 10581047	*Treponema brennaborense DD5/3, DSM 12168*
Gene ID: 10681574	*Treponema primitia ZAS-2*
Gene ID: 2739453	*Treponema denticola ATTC 35405*
Gene ID:10884181	*Treponema paraluiscuniculi Cuniculi A*
Gene ID: 2611411	*Treponema pallidum pallidum Nichols*
Gene ID: 6333710	*Treponema pallidum pallidum SS14*
Gene ID: 10884263	*Treponema paraluiscuniculi Cuniculi A*
Gene ID: 11850998	*Treponema pallidum pallidum DAL-1*
Gene ID: 2739199	*Treponema denticola ATTC 35405*
Gene ID: 10580253	*Treponema brennaborense DD5/3, DSM 12168*
Gene ID: 10681344	*Treponema primitia ZAS-2*
Gene ID: 10884397	*Treponema paraluiscuniculi Cuniculi A*
Gene ID: 2610950	*Treponema pallidum pallidum Nichols*
Gene ID: 6333660	*Treponema pallidum pallidum SS14*
Gene ID: 2739129	*Treponema denticola ATTC 35405*
Gene ID: 10681355	*Treponema primitia ZAS-2*
Gene ID: 10675593	*Treponema azotonutricium ZAS-9*
Gene ID: 10677805	*Treponema azotonutricium ZAS-9*
Gene ID: 10579770	*Treponema brennaborense DD5/3, DSM 12168*
Gene ID: 10677463	*Treponema azotonutricium ZAS-9*
Gene ID: 2740551	*Treponema denticola ATTC 35405*

During the past three decades, and especially since 2004, there have been many reports of antibiotic resistance in treponemes, especially *T. pallidum*. Research studies have confirmed antibiotic resistance associated with macrolides such as erythromycin and azithromycin (12–19). The oral treponemes, *T. denticola*, primarily associated with periodontitis, have shown resistance to tetracycline. The research has also shown that *tetB* and *ermF* genes are now extensively distributed in the *T. denticola* population (20). The most important observation was that three of the *T. denticola* isolates were able to transfer their *ermF* determinants to *Enterococcus faecalis* recipients (20–22). The presence of a natural mobile antibiotic resistance determinant in the genus *Treponema* is very alarming as it could move antibiotic resistant genes between different treponema species. In another study, with *Treponema hyodysenteriae*, intestinal treponemes, 4 of 32 isolates were shown to be resistant to penicillin and produced β-lactamases (23). Mobashery et al. reported penicillin-binding protein Tp47 in *T. pallidum* with an ability of this protein to turn over β-lactam antibiotics. Tp47 is strongly inhibited by products of the β-lactamase reaction, and, therefore, *T. pallidum* remains sensitive to penicillin (24). As the number of cases increase, there could be a potential for mutations in *Tp47* or the presence of a mobile element that can cause multidrug resistance. The present state of knowledge on the diversity of *pbp* genes among clinical and ecological groups of treponemes has not been studied. Examining the natural patterns of occurrence of *pbp* genes in treponemes is an important starting point in understanding how these genes are related to each other and will help to uncover the ecological and evolutionary relationships existing between them.

## Results and discussion

**Fig. 1:**
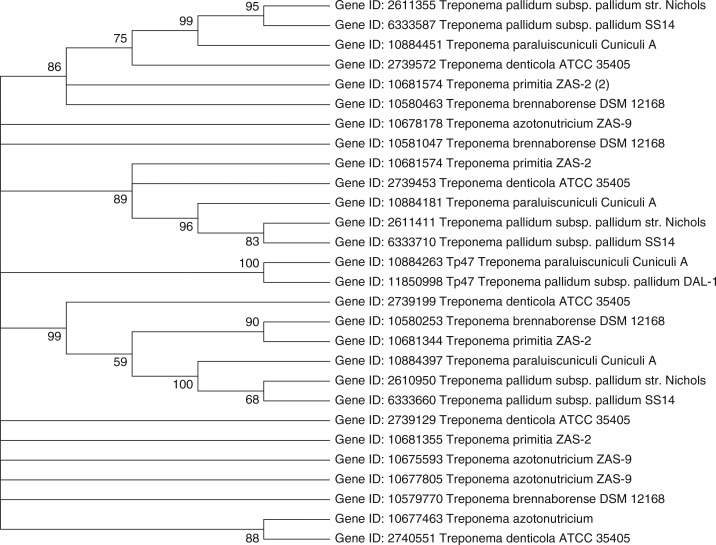
(a) Phylogenetic analysis based on β-lactamase gene sequences from NCBI database. (b) Phylogenetic analysis based on β-lactamase gene sequences constructed after multiple alignment data by CLUSTAL W. (c) The clustering was performed with the maximum likelihood method and Kimura 2-parameter model using the software package MEGA version 5.05 (bootstrap confidence levels are shown as percentages of nodes, consensus tree with values above 50% are shown).

Treponemes are difficult to culture in vitro, a hindrance to experimental approaches such as mutational analysis to identify antibiotic resistance determinants. The report of azithromycin resistance in penicillin allergic patients has emerged as a clinical and public health challenge worldwide. *T. pallidum* continues to be one of the most penicillin-susceptible microorganisms, but mutation in *Tp47* or presence of mobile plasmids with genes encoding β-lactamases could be one of the contributing factors leading to future penicillin resistance in *T. pallidum*. The genome of treponemes, *Treponema azotonutricium* ZAS-9 (25); *Treponema brennaborense* DD5/3, DSM 12168; *T. denticola* ATTC 35405 (26); *T. pallidum* (*pallidum Nichols*) (27); *Treponema pallidum pallidum SS14* (28); *Treponema paraluiscuniculi Cuniculi A* (29); and *T. primitia* ZAS-2 (25), are analyzed for the presence of diversity of *pbp* genes. Phylogenetic calculations using maximum likelihood (ML) methods (30) are shown in Fig. 1. The novel penicillin-binding protein gene *Tp47*, *Treponeme* species (Gene ID 11850998, Gene ID 10884263), forms a separate cluster and does not show substantial similarity to any other *pbp* genes among the *Treponema* species used in this study. The analysis of genome sequence of *T. pallidum* suggests that it lacks genetic elements such as plasmids, bacteriophage, and transposons that are commonly associated with horizontal gene transfer mechanisms. The *pbp* gene for *T. denticola* ATTC 35405 (Gene ID 2739572) has shown 68% similarity to *T. paraluiscuniculi cuniculi A* (Gene ID 10884451) and 65% to *T. brennaborense* DD5/3, DSM 12168 (Gene ID 10580463). *T. paraluiscuniculi* is the causative agent of rabbit venereal spirochetosis (29). *T. denticola*, an oral spirochete associated with periodontal disease, has been reported for the presence of natural mobile antibiotic resistance determinant (20). *T. brennaborense* DD5/3, DSM 12168 (Gene ID 10580463) have shown 68% similarity to *T. pallidum* (*pallidum Nichols*) (Gene ID 2611355). *T. brennaborense* has been isolated from a cow suffering from digital dermatitis. *T. denticola* ATTC 35405 (Gene ID 2739453) has shown 66% similarity to *T. primitia* ZAS-2 (Gene ID 10681574) and *T. paraluiscuniculi Cuniculi A* (Gene ID 10884181). *T. primitia* has been isolated from termite hindguts. *T. azotonutricium* ZAS-9 does not show substantial similarity to any *pbp* genes among treponemes used in this study.

## Experimental section

The list of *pbp* genes for treponemes with complete sequenced genomes was obtained from the NCBI (National Center for Biotechnology Information) database as listed in Tables 1 and 2. The Molecular Evolutionary Genetics Analysis version 5.05 (MEGA5) software program was used for the statistical analyses (30). The BLAST (basic local alignment search tool) algorithm was used to calculate the percentage of similarity between known sequences. The phylogenetic tree was constructed via the ML method, using the Kimura 2-parameter model and a discrete gamma distribution with five categories for capturing non-uniformity of evolutionary rates (K2+G). The K2+G model was selected by virtue of the fact that it had the lowest value of BIC (Bayesian information criterion) (30, 31). One thousand bootstrap trees were generated to determine bootstrap confidence levels (32). The resulting (bootstrap) consensus tree was condensed with values >50% as shown in Fig. 1. The gene sequences used in the study are available for electronic retrieval from the Gene Bank nucleotide sequence database (30).

## Conclusions

The bifunctional *pbp Tp47* had been known for the mechanism of turnover for β-lactam antibiotics in *T. palladum* (24, 33, 34). *Tp47* has showed no substantial similarity to other *pbp* genes in treponemes. Analysis of the *T. pallidum* genome sequence predicted the presence of *pbp* genes. Cha et al. (24) proposed that if a mutant variant of *Tp47* emerges that overcomes the product inhibition of its β-lactamase activity, resistance to penicillin will emerge in *T. pallidum*. However, this requires a multistep mutational process, which is rarer than the single point mutations observed with macrolide resistance. There are no reports of the presence of mobile plasmids in *T. pallidum* although the emergence of a natural mobile antibiotic resistant in *T. denticola* provides no guarantee that it will not move to other *Treponema* species, including *T. pallidum*. The diversity among *pbp* genes across the phylogenetic tree is evident among treponemes that may be representative of their ecological niches. Interestingly, *pbp* gene for *T. denticola* (Gene ID 2739572) has shown relatedness to other treponemes, including *T. paraluiscuniculi Cuniculi A* (Gene ID 10884451); *T. brennaborense* DD5/3, DSM 12168 (Gene ID 10580463); *T. pallidum* (Gene ID 2611355); and *T. primitia* (Gene ID 10681574). Thus, the reported presence of a mobile element in *T. denticola* and the possibility of transfer of antibiotic resistant plasmid may be potentially dangerous, as it could provide a first step toward the acquisition of multidrug resistance. The *pbp* genes for treponemes investigated here could be used for further studies as *Treponema* species are strongly implicated in disease progression. The phylogenetic analysis data presented in this study demonstrate that this organism may possess the potential to acquire antibiotic resistance in the future.
